# Simultaneous Giant Virus and Virophage Quantification Using Droplet Digital PCR

**DOI:** 10.3390/v14051056

**Published:** 2022-05-16

**Authors:** Ana del Arco, Matthias Fischer, Lutz Becks

**Affiliations:** 1 Limnology-Aquatic Ecology and Evolution, Limnological Institute, University of Konstanz, 78464 Konstanz, Germany; lutz.becks@uni-konstanz.de; 2 Department of Biomolecular Mechanisms, Max Planck Institute for Medical Research, 69120 Heidelberg, Germany; matthias.fischer@mpimf-heidelberg.mpg.de

**Keywords:** ddPCR, quantification, giant virus, virophage, CroV, mavirus, cafeteria

## Abstract

Viruses are an abundant component of aquatic systems, but their detection and quantification remain a challenge. Virophages co-replicate with giant viruses in the shared host cell, and can inhibit the production of new giant virus particles, thereby increasing the survival of the infected host population. Here, we present a protocol for Droplet Digital PCR (ddPCR) to quantify simultaneously giant virus and virophage in a mixed sample, enabling the rapid, culture-free and high throughput detection of virus and virophage. As virophage can be present as free virus particles or integrated into the virus host’s genome as well as associated with organic particles, we developed a simple method that enables discrimination between free and particle-associated virophages. The latter include aggregated virophage particles as well as virophage integrated into the host genome. We used, for our experiments, a host-virus-virophage system consisting of *Cafeteria burkhardae*, CroV and mavirus. Our results show that ddPCR can be an efficient method to quantify virus and virophage, and we discuss potential applications of the method for studying ecological and evolutionary processes of virus and virophages.

## 1. Introduction

Viruses are generally considered to be the most abundant biological entities on Earth, with an estimated 5 × 10^31^ particles globally [1]. They shape the dynamics and composition of microbial communities by influencing host mortality and metabolism as well as horizontal gene transfer [2,3,4]. While viruses parasitize their hosts, some complex eukaryotic DNA viruses can be parasitized themselves by smaller viruses called virophages [5,6], which has further consequences for microbial communities and their functions [7]. Only a few cultured systems are available for in-depth studies of protist–giant virus–virophage interactions, such as *Acanthamoeba polyphaga*–mimivirus–Sputnik [8] and *Cafeteria burkhardae*–CroV–mavirus [9], although additional model organisms are on the horizon [10]. Giant viruses are complex viruses with large double stranded (ds) DNA genomes (up to 2500 kb) encoding hundreds of proteins [9,11,12], whereas virophages are small linear or circular dsDNA (17–30 kb) viruses. All virophages described so far are obligate parasites of giant viruses; some of them can also integrate into the host genome where they are transmitted vertically [5,13,14].For the production of progeny virions, the virophage requires the virus factory of the giant virus either during coinfection with the giant virus or following reactivation of the integrated virophage after infection of the host cell with the giant virus [7,15]. As virophages inhibit the production of giant virus particles, and favor host population survival [5,16], host–virus–virophage interactions can play a significant role in natural microbial community dynamics which might vary depending on ecological and environmental conditions.

In the case of *C. burkhardae*, the giant virus CroV and the virophage mavirus, the numbers of viral progeny depend on the mode of infection among other factors. CroV-mavirus coinfection (CroV is inhibited by mavirus) and reactivation (both CroV and mavirus replicate, ~130-CroV particles, ~1000 mavirus particles) of the integrated mavirus virophage lead to the replication and release of infectious mavirus particles [17,18]. Coinfection of host cells with CroV and mavirus leads to the inhibition of CroV replication, as mavirus uses the molecular infrastructure of the cytoplasmic CroV factory for its own replication [5,16]. Individual host cells do not benefit from inhibition of the giant virus, but the decrease in CroV production over time will reduce the number of new infection events and lead to increased host population density. While both giant viruses and virophages are widespread and diverse [14,19,20], their interactions are still poorly understood. For example, the abiotic and biotic conditions that trigger virophage reactivation are still unknown. Understanding whether virophage reactivation or coinfection dominate under specific conditions might help to better predict the community dynamics of microbial systems [18]. In addition, virophage particles can aggregate and bind to debris or to other organisms in the community (e.g., bacteria). Although it is currently unclear how much aggregation influences virion stability and infectivity, bound virophages may have different properties compared to free and integrated virophages. Therefore, quantifying the giant virus, free virophage and particle-associated virophage could help to unravel the role of virophage on both giant virus replication and host population survival under different environmental conditions.

Techniques for virus quantification include plaque assays, flow cytometry, end-point dilution assays, quantitative PCR assays and fluorescence microscopy, but not all methods are applicable to all viruses. Flow cytometry and fluorescence microscopy are culture-independent methods for detecting DNA-containing virus particles, but are limited by viral particle size and genome type [21,22]. Plaque assays, one-step growth curves and end-point dilution assays yield information about infectious virus particles [23], but require that the host of the virus can be cultured. Virophages have small particles (50–75 nm), which makes them difficult to detect with flow cytometry. End-point dilution assays rely on the ability to observe and quantify host lysis. However, because host lysis is reduced in the presence of virophages, and depends on the interaction of the giant virus with the host and of the virophages with the giant virus and host, the results are more difficult to interpret [5]. Furthermore, plaque assays cannot be used with non-platable organisms such as heterotrophic flagellates.

PCR-based methods are independent of virus size and host culturing, but they require knowledge about the viral genome sequence and do not yield information about particles or infectivity. Quantitative PCR (qPCR) provides absolute quantification of a genomic target when used with an appropriate standard of known concentration. Droplet digital PCR (ddPCR) is an alternative that allows absolute nucleic acid quantification by fractionating the sample into multiple parallel PCR reactions. ddPCR detects multiple targets with high specificity and accuracy without having to generate standard curves. The specificity of real-time qPCR can be lowered by the indiscriminate binding of dyes, depending on the total DNA amount and sample background complexity, while ddPCR avoids this type of signal saturation by partitioning the reaction into droplets [24,25]. For example, polysaccharides that are not efficiently removed during DNA extraction can inhibit PCR reactions. Such polysaccharides can stem from bacteria, which are common in cultures of protist hosts. Finally, with real-time qPCR, the dependency between standards and sample quantification may result in noise between sample runs in different plates [26]. ddPCR has been shown to be a repeatable, accurate and rapid quantification method used for basic research with model systems [27,28] as well as in field studies [29]. Here, we assess the potential of ddPCR to quantify simultaneously giant viruses and virophages, and to distinguish between particle-associated and free virophage genomes. We present a protocol for ddPCR detection of the giant virus CroV and the virophage mavirus.

## 2. Materials and Methods

### 2.1. Model System

We used the host *Cafeteria burkhardae* and its viruses Cafeteria roenbergensis virus (CroV) and mavirus. CroV is a giant virus with a particle diameter of 300 nm and a genome size of 692 kb [9]. The virophage mavirus has a particle size of ~70 nm and a small linear DNA genome (~30 kb) [5]. For DNA extractions and subsequent ddPCR, we used stock cultures containing the giant virus or the virophage.

### 2.2. DNA Extraction

For ddPCR assays, DNA was extracted using the DNeasy 96 Blood & Tissue Kit (Qiagen, Hilden, Germany). Specifically, we used the Blood and Body Fluid Spin Protocol with the following modifications: (1) sample incubation in lysis buffer: 100 µL sample, 10 µL Proteinase K, 100 µL AL buffer and without RNase treatment. (2) elution: 100 µL AE buffer. DNA concentrations were measured using a NanoDrop ND-1000 Spectrophotometer (Thermo Fisher Scientific, Wilmington, DE, USA) and DNA samples were kept at 4 °C until used for ddPCR. We used the same DNA samples for all optimization tests.

### 2.3. Evaluation of Primer and Probe Specificities in Singleplex and Multiplex ddPCR

We designed primers and probes for virus DNA amplification (Table 1) following the Droplet Digital PCR Applications Guide (https://www.bio-rad.com/webroot/web/pdf/lsr/literature/Bulletin_6407.pdf accessed on 20 April 2020) and based on a previous study on the use of ddPCR for experimental evolution studies [27]. The probe for mavirus was labeled with FAM as a fluorescent dye, the probe for CroV with HEX. We tested the primer and probe specificities by running singleplex and multiplex tests. Before the PCR step, the template DNA, primer, probes, and the PCR master mix (ddPCR Multiplex Supermix, BioRad, Feldkirchen, Germany) were mixed. Next, the mix was used to generate droplets (QX200 droplet generator, BioRad), and the droplets were then used in the PCR reaction. After the PCR step, the PCR products were read using a QX200 Droplet Reader (BioRad, Feldkirchen, Germany). Our singleplex and multiplex tests included: (1) virophage DNA with virophage specific primers and probes, (2) virophage DNA with giant virus specific primers and probes, (3) giant virus DNA with virophage specific primers and probes, (4) giant virus DNA with giant virus specific primers and probes, and (5) giant virus and virophage DNA with both pairs of primers and probes (for all tests: number of technical replicates *n* = 6). For specificity tests, we used an annealing temperature of 58 °C, 10 pg µL^−1^ as the DNA template. For primers and probe concentrations, we followed the recommended concentrations for the multiplex supermix (Table 2). PCR parameters were 1 cycle of 95 °C (10 min), 40 cycles of 94 °C (30 s), 40 cycles of 58 °C (1 min) and 1 cycle of 98 °C (10 min). We did not use a digestion enzyme prior to ddPCR, due to the small genome size of the giant virus and the low DNA concentrations used.

All ddPCR results were analyzed using the QUANTASOFT 1.7.4 software, where counts displayed in channel 1 (FAM, blue color) and channel 2 (HEX, green color) represented the virophage and the giant virus, respectively. Count values originated from the thousands of droplets generated in the droplet generator, and each droplet represented an individual PCR reaction that contained (positive count) or did not contain (negative count) the target DNA. QUANTASOFT provides 1D and 2D-scatter plots, information on the ratio of the counts from channel 1/channel 2 and events corresponding to droplets counts (total, positive and negative), and uses this information to estimate DNA concentrations in the samples. To evaluate the quality of the quantification, 1D-scatter plots can be used to visualize positive and negative counts and to detect “rain”, which means droplets that could not be allocated as positive or negative, and “heavy rain”, which indicates poor quantification because of poor target separation. The latter is due to high DNA concentrations of the templates and/or inadequate annealing temperature. 2D-scatter plots represent intensity of the fluorescence for channel 1 (FAM) plotted against fluorescence for channel 2 (HEX). They can be used to visualize positive droplets for either channel (FAM in blue; HEX in green), double negative (neither FAM and HEX, grey) and double positives (both FAM and HEX, orange). With too few droplets (<10,000), a precise application of the Poisson statistics used by the software for droplets separation is not possible, and the samples cannot by analyzed.

### 2.4. Optimization of PCR Conditions and DNA Concentrations

We ran a thermal gradient to evaluate the optimal annealing temperature. The temperature gradient (number of technical replicates for each temperature *n* = 3) ranged from 64 °C to 54 °C using the same DNA and primer/probe concentrations as for the single- and multiplex tests (10 pg µL^−1^). We further tested different concentrations of DNA templates for giant virus and virophage (10 pg µL^−^^1^, 1 pg µL^−1^ and 0.1 pg µL^−1^; number of technical replicates for each template *n* = 6) to determine the best range for quantification accuracy. DNA concentrations were selected based on previous preliminary experiments. We used the primer concentrations recommended by BioRad© for the ddPCR Multiplex Supermix (Table 2).

### 2.5. Discrimination between Free and Particle Associated Virophage

We used the optimized multiplex protocol to test if the number of particle-associated virophages could be calculated based on the difference between free virophage and total virophage present in the samples. We used free virophages from a stock (see above) in combination with three different host strains. The host strains differed with regard to the presence or absence of integrated mavirus. *Cafeteria burkhardae* strains E4-10P and RCC970E3 were used as mavirus-negative controls, although recent studies (Fischer, et al., unpublished) revealed that the strain E4-10P will occasionally produce mavirus-like particles in response to CroV infection. However, in the experiments described here, we did not detect any mavirus-specific signal in uninfected or CroV-infected E4-10P or RCC970E3 cells. RCC970E3-8.8 had two mavirus insertions and was derived from strain RCC970E3. We combined each strain (1450 µL, ~10^5^ cell mL^−1^) with the virophage from the stock (50 µL, 14,000 virophage particles mL^−1^). We had three treatments each with four replicates per host strain: Control (C): host with no virophage added; Total Virophage (T-V): host and added virophage; Free Virophage (F-V): filtrate of samples with host and added virophage. We used 200 µL samples directly taken from the host and virus mixes for DNA extraction for the C and T-V treatments. For the F-V treatment, we first filtered the samples through a 0.1 µm sterile cellulose syringe filter and used 200 µL of the filtrate for DNA extractions. Filtration removes host cells and particle-associated virophage. The latter includes integrated virophage, aggregates of virophage or virophage attached to bacteria or cell debris as well as some of the free virophage particles. Finally, 200 µL of each replicate for all treatments were used for DNA extractions. We used these samples to estimate the fraction of particle-associated virophage by subtracting the virophage concentration in the filtrate (F-V treatment) from the virophage concentration in the unfiltered sample (T-V treatment). We further used the samples to evaluate the removal of virophage through filtration by calculating the difference in virophage concentration between the unfiltered sample and the filtered samples from strains without integrated virophage.

## 3. Results

### 3.1. Specificity of Primers and Probes in Singleplex and Multiplex ddPCR

**Figure 1:**
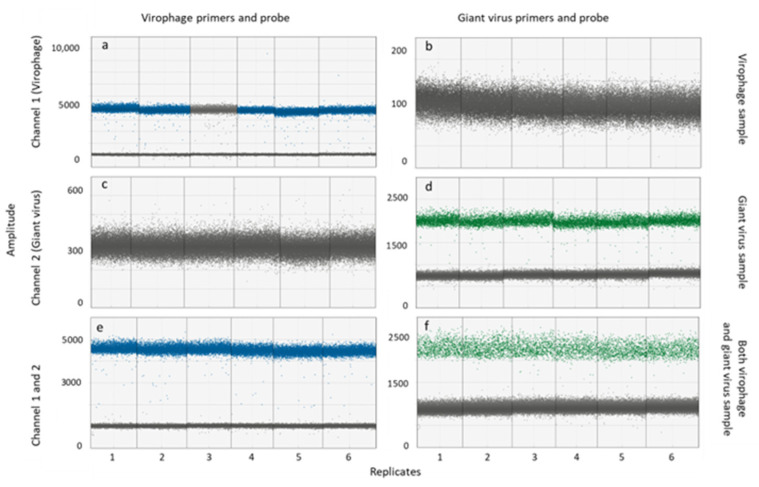
Summary of Quantasoft analyses for 1D-scatter plots of droplet fluorescence for both virophage and giant virus in singleplex and multiplex assays (technical replicates *n* = 6). (**a**–**d**) The singleplex test shows only the giant virus or the virophage present (right y-axis) with primers and probes for the virophage (left column) or the giant virus (right column). The giant virus and the virophage only amplified with their specific sets of primers and probes (**a**,**d**) but not vice versa (**b**,**c**). Both virus DNA targets were amplified in the multiplex test by giant virus and virophage DNA templates and primers and probes for the virophage (**e**) or the giant virus (**f**).

**Figure 2:**
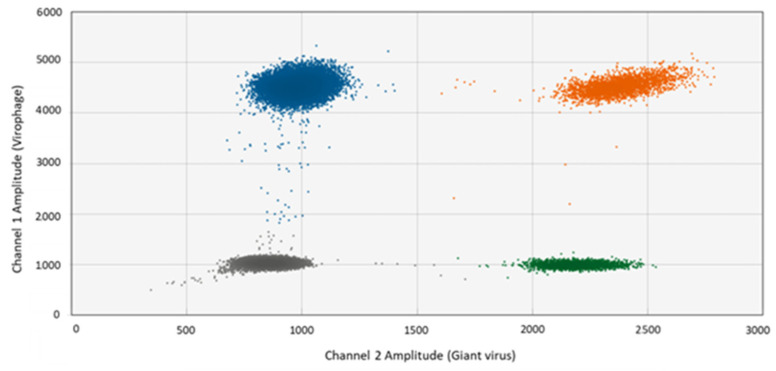
2D-scatter plot of a multiplex ddPCR experiment containing giant virus and virophage templates with their primers and probes (see Figure 1e,f). Channel 1 shows FAM fluorescence (virophage, blue) and Channel 2 shows HEX fluorescence (giant virus, green). Double negatives are shown in grey, double positives in orange.

The ddPCR method is based on the generation of thousands of monodisperse droplets in which the PCR reactions take place. Each droplet has a probe-specific fluorescence signal that depends on the presence or absence of the DNA template. Therefore, each droplet is rated positive or negative depending on the fluorescence amplitude. On this basis, the fraction of positive droplets representing the target DNA sequence is calculated as DNA copies mL^−1^ based on the known total amount of droplets generated. Results from singleplex and multiplex tests confirmed that the primers amplified the target without cross-amplification, and that the probes annealed only to giant virus or to virophage templates, respectively, (Figure 1a–d and Figure 2). When the giant virus and virophage templates were mixed, amplification was successful, as indicated by band separation (Figure 1e,f). Specifically, blue and green bands represent the positive droplet signal for the giant virus and the virophage, respectively, while grey bands represent the negative droplet with no fluorescence signal. We used 2D-scatter plots (Figure 2) to evaluate the multiplex test, and found that the droplets separated into four groups: droplets containing either virophage or giant virus amplicons, droplets containing both amplicons, and droplets without amplicons. Thus, the primers and probes were giant virus- and virophage-specific when multiplexed, and cloud separation in the 2D-scatter plots enabled the quantification of the giant virus and of the virophage.

### 3.2. PCR and DNA Conditions

**Figure 3:**
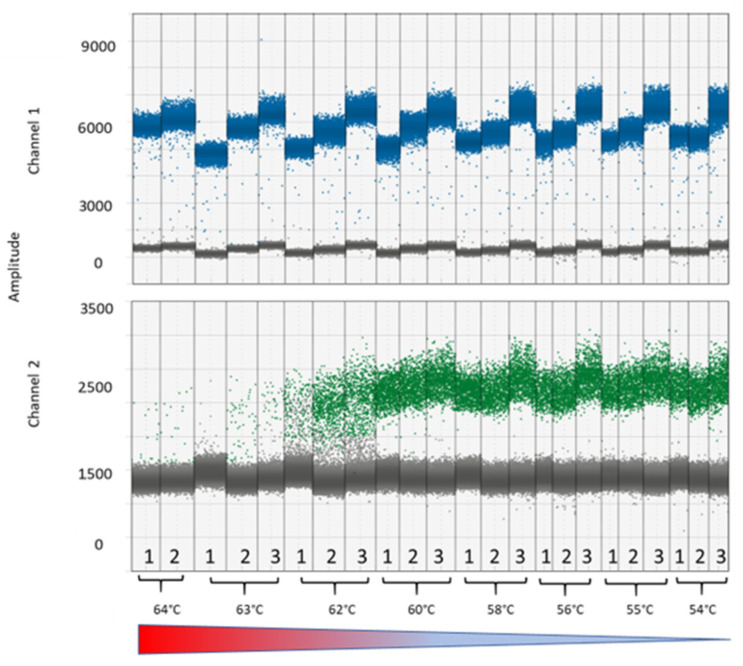
Thermal gradient PCR for optimizing the annealing temperature with temperatures ranging from 64 °C to 54 °C. Gradients were used for the giant virus and virophage primers and probes (technical replicates *n* = 3, one technical replicate was lost for temperature 64 °C).

We found that the virophage signals separated well when using annealing temperatures (T_ann_) between 54 °C and 64 °C, while band separation for the giant virus occurred only at T_ann_ < 62 °C (Figure 3). Average counts in the T_ann_ range where band separation was observed for both virus and virophage were 53.9 ± 1.0 virophage copies µL^−1^ and 7363 ± 203 giant virus copies µL^−1^, indicating similar counts across temperatures. Count differences among the replicates were related to sample preparation, which was done in cartridges where eight samples at a time could be prepared. Each replicate was prepared in a different cartridge, which could explain the variation in counts. We chose T_ann_ = 58 °C for the following assays, as band separation for both the giant virus and the virophage was good at this temperature, and higher temperatures minimized unspecific PCR amplification.

**Figure 4:**
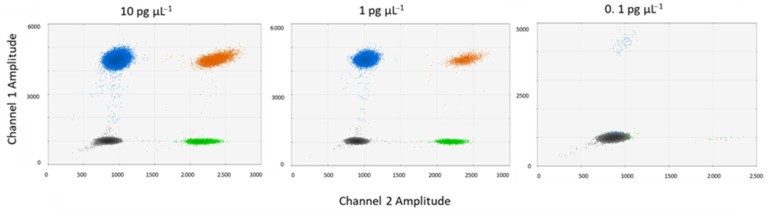
Effect of template DNA concentration on ddPCR quantification. Shown are 2D-scatter plots for three different template DNA concentrations containing both giant virus and virophage DNA at equal concentrations (three technical replicates). For color schemes see Figure 2.

Testing for the optimal DNA template concentration, we found that 1 pg µL^−1^ resulted in optimal droplet separation and lower double positive droplets where both targets were detected simultaneously (orange cloud, ~2% of total droplets) (Figure 4). The higher DNA concentration (10 pg µL^−1^) also showed good band separation, but the number of double positive droplets was higher (~5% of total droplets) compared to the intermediate concentration (~2% of the droplets). DNA concentrations of 0.1 pg µL^−1^ were too low to produce sufficient positive counts for accurate quantification. Both 1 pg µL^−1^ and 10 pg µL^−1^ DNA template concentrations provided good band separation, therefore DNA concentrations within this range are suitable for quantification. However, we choose 1 pg µL^−1^ as the optimal concentration to prevent oversaturation of DNA concentration and compromised band separation.

### 3.3. Discrimination between Free and Particle-Associated Virophages

**Figure 5:**
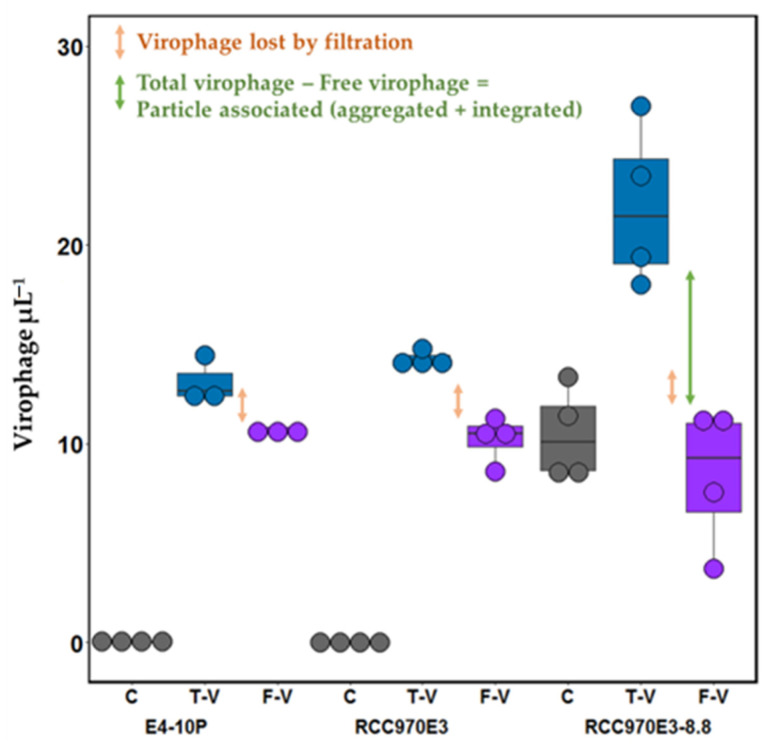
Virophage ddPCR-based quantification. Shown are copy number concentrations (virophage µL^−1^; median, 4 replicates) for host strains without integrated virophage (E4-10P, RCC970E3) and with integrated virophage (RCC970E3-8.8). Control (C; grey): host strain with no virophage added, no filtration before DNA extraction; Total-Virophage (T-V, blue): host and virophage, no filtration before DNA extraction; Free-Virophage (F-V, purple): filtered sample of host and virophage before DNA extraction.

We found that 0.1 µm pore-size filtration of samples resulted in removal of 19% and 24% of free virophage in E4-10P and RCC970E3, respectively, (Figure 5, orange arrows). Using the mavirus-containing *C. burkhardae* strain RCC970E3-8.8, we explored the possibility of discriminating between free and particle-associated virophages. The overall virophage densities of RCC970E3-8.8 from the control (Figure 5, RCC970E3-8.8 grey) and free-virophage treatment (Figure 5, RCC970E3-8.8 purple) were comparable to the virophage density detected in the total-virophage treatment (Figure 5, RCC970E3-8.8 blue), which contained both the host strain with integrated virophages in the same density as the control, and the same amount of added free virophage as in the free-virophage treatment. However, a loss of 18% was observed (Figure 5, orange arrow), likely due to mavirus retention on 0.1 µm pore-size filters. Quantification of the different virophage fractions allowed the estimation of the particle-associated virophage (Figure 5, green arrow).

## 4. Discussion

We tested multiplex ddPCR for the quantification of a giant virus and a virophage in mixed samples of CroV and mavirus, and found that this method enables discrimination between giant virus and virophage. Using an optimized ddPCR protocol for multiplexing, we were able to measure and quantify the differences between the total and free virophage fraction in experimental samples, and thus estimate how many virophages may be integrated in a host genome and/or bound to particles larger than 100 nm. We observed that about 20% of mavirus genomes were removed from a sample after filtration with a 0.1 µm pore-size syringe filter. Target losses may be associated with the filter material, which could be a common issue for other systems. Therefore, the protocol could be improved by identifying filters that reduce the losses. The filtration approach used here does not discriminate between integrated virophage genomes, genomes in free virions, or genomes attached to larger particles. Separating the integrated from the particle-associated virophages would require additional protocol development. Quantitative PCR methods can detect single copies of virus and virophage DNA. ddPCR thus quantifies virophage DNA integrated into the host genome and inside virions, whether they be free or bound to organic particles, other virus particles, or debris. In contrast, one-step growth curves cannot detect single copies within aggregates or when integrated into the host without separating virophages from each other, the host or particles. In addition, plaque assays cannot be used to quantify virophages, as virophages are dependent on viral replication and the non-plateable behaviour of the host.

We have shown that multiplex ddPCR allows simultaneous and detection quantification of giant virus and virophage targets, which can be used as a tool to explore the ecological and evolutionary dynamics of these viruses in mixed samples. For example, the inhibitory effect of mavirus on CroV replication differs between coinfection events with free virophage particles and reactivation events from host-integrated virophage genomes [16]. Being able to quantify the fraction of integrated vs. free virophage is important in order to understand the coevolutionary dynamics of this and related systems. While periods of coinfection of virophage and virus can accelerate coevolution, as in other antagonistic systems [3,30], periods with mainly horizontal transmission might slow down coevolution or select for different traits of giant virus and virophage. Furthermore, multiplex ddPCR can be used for exploring evolutionary changes, such as the evolution of virulence or resistance in the interaction between giant virus and virophage over time. Specifically, different isolated populations of giant virus and virophage stemming from different time points or locations of sampling, can be used to assess how giant virus-virophage interaction changed (i.e., quantifying and comparing virion production from combinations of different isolates) [3], such as is done using flow cytometry for assaying virus life-history traits (e.g., [31]). In the context of experimental evolution studies, comparison of virus populations isolated from the experiments with ancestral populations (i.e., the virus population that was used to inoculate the experiments) permits measurement of the population average trait changes (e.g., [32]). Changes in the main trait values (e.g., replication, virulence) of the population in comparison to ancestral populations would indicate evolutionary changes in the system (e.g., changes in genotype frequencies, new genotypes by de novo mutations). ddPCR is a highly sensitive method that allows for the simultaneous detection of giant virus and virophage even at low densities and ratios (e.g., other studies detect target differences in mixed samples with ratios of 0.001 [27]). Thus, differences in the ration of giant virus and virophage production could be detected, and evolutionary changes in quantitative traits could be studied. This includes the quantification of virophage and giant virus replication. The latter trait is predicted to evolve towards lower replication because it lowers the level of the exploitation by the virophage [33]. The two main advantages of ddPCR over other quantitative PCR methods are droplet partitioning, which leads to parallel PCR reactions, and the fact that no standard curve is required. However, the cost of reagents and ddPCR equipment should be considered [27].

Giant viruses and virophages are globally abundant and widespread [34], and their interaction can shape microbial communities [5,6,35]. However, the temporal dynamics of host, virus and virophage populations are not known. ddPCR and multiplexing allowed us to detect and quantify giant virus and virophage in mixed samples, and in combination with a filtration step to discriminate between free and particle-associated virophage. The dominance of free or particle-associated virophage will promote different modes of infection, and diverse modes of infections have the potential to result in different population dynamics [18]. As there is an increasing interest in the ecological and evolutionary dynamics in tripartite systems in microbial communities such as hosts, helper viruses and satellite viruses, the presented methods for multiplex ddPCR should be considered for other systems as well. This method can help the study of the role of virophage-giant virus interactions, and has the potential to be used for a wide range of disciplines, for clinical samples to identify drug-resistant subpopulations [36], agricultural evaluation of pest resistance [37], and field surveying of viruses [29] among others.

## Figures and Tables

**Table 1:** Primers and probes.

Name	Description	Sequence (5′ to 3′)
Mavirus_forward	Forward primer	GAATGTCTCGCGGTTTAGGT
Mavirus _reverse	Reverse primer	TGGCTACAAGTGCTTCATCTAC
Mavirus _probe	FAM Probe (Ch 1)	56-FAM/ATTATATCCACCCACGGGCAGCAG/3BHQ_1
CroV_forward	Forward primer	GAAACTGGAAATGCTCGTGTTAT
CroV_reverse	Reverse primer	GGGAAAGAACCTGGTCGTAATAC
CroV_probe	HEX Probe (Ch 2)	5HEX/ACTGCAACACCTGCAATCAATCAACC/3BHQ_1

**Table 2:** PCR parameters for virus quantification.

Parameter	Tested Range	Optimal Value
PCR annealing temperature	64–54 °C	58 °C
DNA template	0.1–1–10 pg µL^−1^	1 pg µL^−1^
Primer/Probe CroV	Recommended concentrations	Primer 375 nM Probe 125 nM
Primer/Probe mavirus	Recommended concentrations	Primer 375 nM Probe 125 nM

## Data Availability

The datasets used and/or analyzed during the current study are available from the corresponding author on request.
